# Primary Diffuse Large B‐Cell Lymphoma of the Female Genital Tract: A Rare Case Presentation With Literature Review

**DOI:** 10.1155/crh/7682047

**Published:** 2026-09-29

**Authors:** Feifei Xu, Jie Lin Jaslyn Lee, Baozhu Yi, Wanwan Chen

**Affiliations:** ^1^ Department of Pathology, The Second Affiliated Hospital of Wenzhou Medical University, Wenzhou, Zhejiang, China, wmu.edu.cn; ^2^ Department of Pathology, National University Hospital, Singapore, nuh.com.sg

**Keywords:** female genital tract, gene mutation, primary diffuse large B-cell lymphoma

## Abstract

Primary lymphoma of the female genital tract is a rare entity with nonspecific clinical manifestations, rendering accurate diagnosis challenging. We describe an unusual case of primary diffuse large B‐cell lymphoma (DLBCL) of the female genital tract. The patient presented with abdominal distention and difficulty breathing. A total hysterectomy, bilateral salpingo‐oophorectomy, and omentectomy were subsequently performed, and the final histology was a DLBCL of germinal center subtype (GCB), with mutations in *TP53* and *MYC* genes. The patient received attenuated dose chemotherapy with rituximab, cyclophosphamide, doxorubicin, vincristine, and prednisolone (R‐CHOP), 3 cycles every 3 weeks. She died 3 months after surgery. This case highlights the aggressive disease biology of GCB‐type DLBCL with *TP53* and *MYC* gene mutations.

## 1. Introduction

Lymphoma originating from the female genital tract is rare, with the ovary and cervix representing the most frequent sites of involvement [1]. Distinguishing between primary and secondary lymphoma can be difficult, especially in cases with concurrent nodal and extranodal disease. An accurate diagnosis is crucial for proper treatment and prognosis [2, 3]. We describe here a rare case of primary DLBCL of the female genital tract. We also present a review of the relevant scientific literature.

## 2. Case Report

A 67‐year‐old postmenopausal woman presented with a one‐month duration of abdominal distention and difficulty breathing. An initial transvaginal ultrasound (TVUS) demonstrated an enlarged uterus, fluid in the uterine cavity, and bilateral hypoechoic adnexal masses. Magnetic resonance imaging (MRI) demonstrated enlarged uterus and bilateral adnexal masses measuring 40 × 35 mm (left) and 23 × 21 mm (right), accompanied by extensive thickening of the mesentery, peritoneum, and bladder upper wall (Figure 1). Human papillomavirus (HPV) testing and cervical cytology were negative. Cytology of the peritoneal washing showed atypical lymphocytes. Subsequently, the patient underwent surgery to remove masses. Intraoperatively, the uterus was enlarged to approximately 12 weeks pregnant size and adherent to the bladder. There were multiple omental and peritoneal nodules measuring up to 4 cm in size, with extensive bowel and omental adhesions resulting in a frozen pelvis. The intraoperative frozen sections showed a malignant small round cell tumor, and lymphoma could not be excluded. Considering the large volume and wide range of involvement of the tumor, the patient had already experienced severe nerve compression pain. Therefore, in order to alleviate symptoms, a total abdominal hysterectomy, bilateral salpingo‐oophorectomy, intestinal adhesiolysis, and omentectomy were performed. On a gross examination, the uterus was diffusely enlarged and slightly soft, but no discrete mass lesions were noted (Figure 2). Paraffin sections showed diffuse sheets of large malignant cells with occasional prominent nucleoli, coarse chromatin, and increased mitotic activity (Figure 3). The tumor involved the uterus (full‐thickness myometrial involvement), cervix, bilateral adnexal, and bilateral uterine horns, accompanied by invasion of the omentum and peritoneum. On immunohistochemistry, the tumor cells stained positive for CD20, c‐Myc, and Bcl‐6, while negative for CD10, Bcl‐2, and MUM‐1. The Ki‐67 proliferative index was more than 95% (Figure 4). Fluorescence in situ hybridization (FISH) was used to detect *MYC*, *BCL2*, and *BCL6* genes and demonstrated no gene rearrangements. Next‐generation sequencing (NGS) of the tumor showed mutations in *TP53* on Exon 8 c.817C > T p. Arg 273Cys (40.30%) and Exon 5 c.535C > T p. His179Tyr (44.04%), as well as a mutation in *MYC* on Exon 2 c.220C > G p. Pro74Ala (63.28%). The final diagnosis was a GCB‐type DLBCL with TP53 and MYC gene mutations. Given the patient’s lesion characteristics met the following criteria, she was diagnosed with primary lymphoma of the female genital tract. (1) The lesion was clinically confined to the female genital tract, involving the peritoneum; (2) bone marrow and peripheral blood analyses revealed no abnormalities; and (3) within 2 months of initial diagnosis, new lesions only appeared in the bilateral adnexal regions.

**FIGURE 1:**
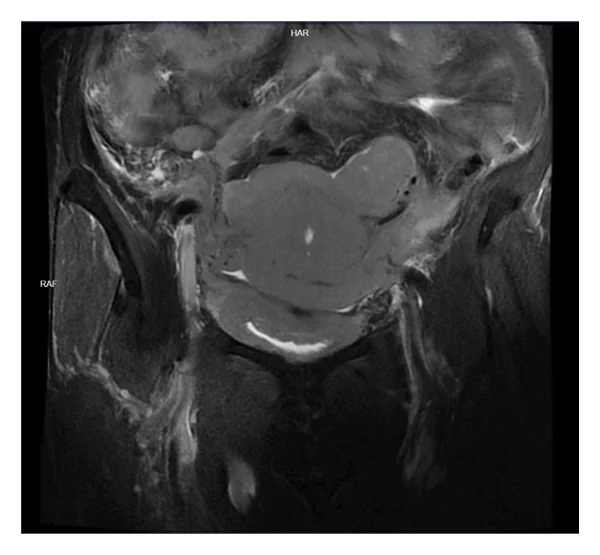
MRI demonstrating diffuse uterine enlargement and adnexal masses.

**FIGURE 2:**
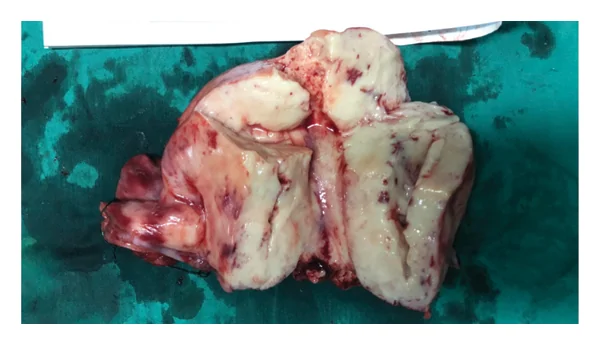
The gross specimen showing the enlarged uterus and right adnexa.

**FIGURE 3:**
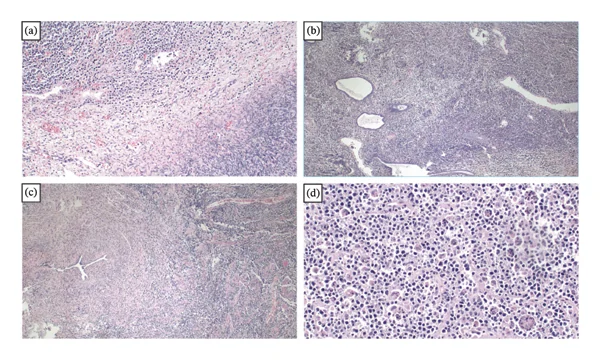
Low power view of (a) ovarian stroma, (b) endometrium, and (c) fallopian tubes showing tumor cells with a diffusely infiltrative growth pattern. (d) High power view of the tumor cells showing significant nuclear atypia with prominent mitotic activity.

**FIGURE 4:**
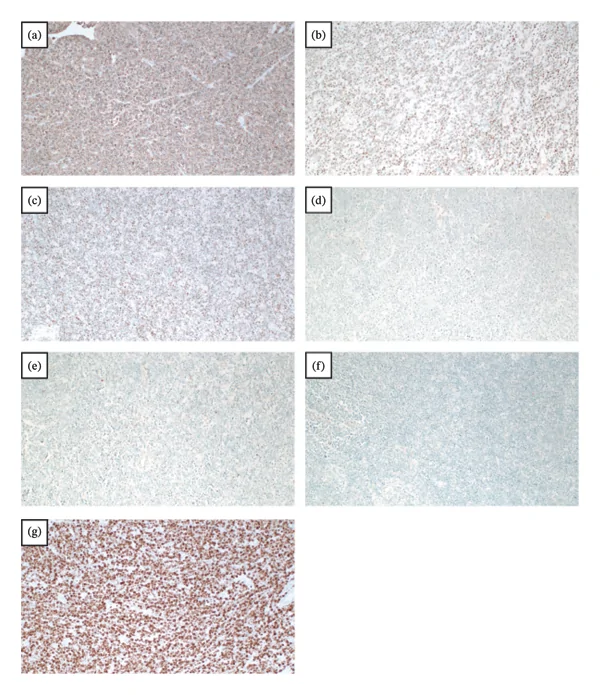
Immunohistochemistry showing positive results for (a) CD20, (b) c‐Myc, and (c) Bcl‐6, while negative results for (d) CD10, (e) Bcl‐2, and (f) MUM‐1. The proliferation index for (g) Ki67 exceeding 95%.

The patient was scheduled for six cycles of chemotherapy but completed only three cycles due to rapid disease progression. R‐CHOP was administered at attenuated doses, specifically with doxorubicin at 30 mg/m^2^. No further dose reductions or treatment delays occurred. Two months after surgery, the patient underwent a follow‐up examination. Laboratory evaluation showed lymphocytes 0.391 × 10^9^/L, leukocytes 5.58 × 10^9^/L, platelets 258 × 10^9^/L, and hemoglobin 86 g/L. Chemistry revealed creatinine 44 μmol/L (normal range: 46–92 μmol/L), urea nitrogen 11.49 mmol/L (normal range: 2.5–6.1 U/L), and LDH 2498 U/L (normal range: 120–250 U/L). Computed tomography (CT) revealed new lesions in both adnexal regions. Unfortunately, she did not respond well and passed away 3 months after surgery. Her cause of death may be related to multiple organ failure caused by a large tumor burden and a wide range of involvement, large surgical scope, and poor chemotherapy response.

## 3. Discussion

Primary female reproductive tract lymphoma is a rare and easily misdiagnosed disease, with the most common histologic subtype being DLBCL. Patients generally present with symptoms typical of conventional female genital tract malignancies, such as vaginal bleeding, abdominal pain, and often lack classical systemic symptoms associated with lymphoma, such as fatigue, fever, weight loss, and night sweats [4]. Our patient presented without specific lymphoma symptoms, but the radiological findings of diffuse uterine enlargement and bilateral adnexal masses raised the possibility of a lymphoma.

Due to the limitations of the frozen section, it is challenging to confirm the diagnosis of lymphoma and almost impossible to determine the lymphoma type intraoperatively. Pathologists often report such cases as malignant small round cell tumor, deferring definitive diagnosis to paraffin sections [5]. Integrations of histological and immunohistochemical findings are required to arrive at a diagnosis of DLBCL. On histology, DLBCL usually shows diffuse sheets of medium‐sized to large lymphoid cells with vesicular nuclei, coarse chromatin, scant cytoplasm, and brisk mitotic activity. Immunohistochemistry of DLBCL demonstrates positivity for markers such as CD20, PAX‐5, MUM‐1, and a high Ki67 proliferative index [6]. The Hans algorithm divides DLBCL cases into two groups (GCB and non‐GCB subtypes) according to immunohistochemical expression of CD10, Bcl‐6, and MUM‐1. The prognosis of non‐GCB subtype of DLBCL is significantly worse than that of GCB‐subtype [7]. In our case, the tumor cells were positive for Bcl‐6 and negative for MUM‐1, which is consistent with a GCB subtype.

Comprehensive genetic testing (such as NGS and FISH) can provide accurate molecular typing for DLBCL, which is beneficial for achieving personalized treatment and prognosis judgment. *TP53* mutation is a marker for poor prognosis of DLBCL, which endows tumors with chemotherapy resistance. *MYC* mutation is a carcinogenic driving mechanism that does not rely on rearrangement, directly promoting tumor proliferation. These two events often work together, defining a type of “true double mutation” high‐grade lymphoma in this area, with extremely high invasiveness and recurrence risk, and may be resistant to the R‐CHOP regimen [8]. Our patient was diagnosed with GCB‐subtype DLBCL. FISH analysis showed no rearrangements in *MYC*, *BCL2*, or *BCL6*. However, NGS testing identified mutations in both *TP53* and *MYC* genes. She received attenuated dose chemotherapy after surgery timely but died three months after surgery, indicating that GCB‐subtype DLBCL with *TP53* and *MYC* gene mutations is highly aggressive. Therefore, in the context of DLBCL of GCB subtype with no rearrangements in *MYC*, *BCL2*, or *BCL6*, further detection of *TP53* and *MYC* mutations by NGS is of great significance.

While diagnosing DLBCL, it is critical to distinguish primary and secondary disease. The diagnostic criteria for primary lymphoma of the female genital tract are as follows: (1) the disease is limited to the female genital tract, (2) there is no evidence of disease elsewhere in the body after full examination, (3) no tumor cells are found in the peripheral blood or bone marrow, and (4) there is no previous history of lymphoproliferative disorder [9]. If the patient meets these criteria for primary lymphoma of the female genital tract, staging follows the Ann Arbor classification [10].

Primary DLBCL of the female genital tract is generally treated with chemotherapy [11]. Most patients can benefit from the standard chemotherapy regimen of R‐CHOP, while the rest may have a poor prognosis due to tumor resistance or relapse [12]. For highly aggressive B‐cell lymphoma, the combination of DA‐EPOCH‐R (dose‐adjusted etoposide, prednisone, vincristine, cyclophosphamide, doxorubicin, and rituximab) with targeted drugs may show additional superiority over the R‐CHOP [13]. Surgery in patients with gynecological lymphoma should be performed solely to obtain tissue for pathological diagnosis, thereby guiding subsequent radiotherapy and chemotherapy. However, surgery is typically required to remove a mass when no initial suspicion of lymphoid proliferative disease exists, which may compromise the patient’s physical state. This phenomenon likely reflects the invasive biological behavior of DLBCL. We have recorded a short table summarizing several previously reported primary female genital tract DLBCL cases (Table 1).

**TABLE 1:** Literature review of previously reported primary female genital tract DLBCL cases.

Author	Age (year)	Site	Stage	Treatment	Outcome
Novotny et al. [14]	82	Cervix	I	None	Died
Goda et al. [4]	52	Cervix	IAE	R‐CHOP + ISRT	CR
Goda et al. [4]	50	Cervix	IE	R‐CHOP + IFRT	CR
Goda et al. [4]	39	Cervix, vagina, uterine segment	IAEX	R‐CHOP + ISRT	CR
Goda et al. [4]	62	Cervix	IIAE	R‐CEOP + IFRT	CR
Du et al. [15]	48	Endometrium	IE	Surgery + R‐CHOP	CR
Parva et al. [16]	21	Cervix	IEA	R‐CHOP	CR
Baijal et al. [17]	44	Cervix	IE	R‐CEOP + IFRT	CR

*Note:* IFRT, involved field radiotherapy; ISRT, involved site radiotherapy; R‐CHOP, rituximab with cyclophosphamide, adriamycin, vincristine, and prednisalone; R‐CEOP, rituximab with cyclophosphamide, etoposide, vincristine, and prednisolone.

Abbreviation: CR, complete response.

## 4. Conclusion

This report describes an unusual clinical case of GCB‐subtype DLBCL, harboring *TP53* and *MYC* mutations in the absence of *MYC*, *BCL2*, or *BCL6* rearrangements. The patient died 3 months after undergoing a large‐scale surgery, prompting clinicians to reflect on and learn from this unfortunate outcome. For large masses suspected of being lymphoma, diagnostic puncture is preferable to radical resection, except when the purpose is to relieve mass‐related compression symptoms. While diagnosing DLBCL, it is recommended to perform FISH testing for *MYC*, *BCL2*, and *BCL6* rearrangements, along with NGS testing for related genes. This method identifies patients belonging to a high‐risk subgroup, a classification that directly informs subsequent treatment planning.

## Author Contributions

Feifei Xu carried out the initial drafting of the manuscript, Baozhu Yi provided the MRI image and reviewed the manuscript, and Jie Lin Jaslyn Lee and Wanwan Chen reviewed the manuscript.

## Funding

This study was supported by the Natural Science Foundation of Zhejiang Province (LQ22H040008) and Wenzhou Municipal Scientific Research (Y2023509).

## Ethics Statement

This study was approved by the local ethics committee.

## Consent

The local ethics committee granted a waiver for informed consent (2024‐K‐368‐01). All personally identifiable patient information has been removed from this article.

## Conflicts of Interest

The authors declare no conflicts of interest.

## Supporting Information

Additional supporting information can be found online in the Supporting Information section.

## Supporting information


**Supporting Information** 2013 CARE Checklist.

## Data Availability

All data relevant to this case report are included in the article.
