# Supplementary material for: Primary Diffuse Large B‐Cell Lymphoma of the Female Genital Tract: A Rare Case Presentation With Literature Review
Source: Case Rep Hematol. 2026 Sep 29;2026:7682047. doi: 10.1155/crh/7682047 (PMC13624386; doi:10.1155/crh/7682047)
Supplement: Supplementary file 1 [file CRH-2026-7682047-s001.docx]

**2013 CARE Checklist**

1. **Title** – The diagnosis or intervention of primary focus followed by the words “case report”. Page1, Lines1-2
2. **Key Words** – 2 to 5 key words that identify diagnoses or interventions in this case report (including "case report"). Page1, Line20
3. **Abstract** – (structured or unstructured)

Introduction – What is unique about this case and what does it add to the scientific literature? Page1, Lines17-18

The patient’s main concerns and important clinical findings.

Page1, Line12

The primary diagnoses, interventions, and outcomes. Page1, Lines12-17

Conclusion – What are one or more “take-away” lessons from this case report? Page1, Lines17-18

4. **Introduction** – Briefly summarizes why this case is unique and may include medical literature references. Page1, Lines30-31
5. **Patient Information**
   - De-identified patient specific information. Not mentioned
   - Primary concerns and symptoms of the patient. Page1, Lines34-35
   - Medical, family, and psychosocial history including relevant genetic information. Not mentioned
   - Relevant past interventions and their outcomes. Not mentioned
6. **Clinical Findings** – Describe significant physical examination (PE) and important clinical findings. Page1, Lines34-35
7. **Timeline** – Historical and current information from this episode of care organized as a timeline (figure or table). Not mentioned
8. **Diagnostic Assessment**
   - Diagnostic methods (PE, laboratory testing, imaging, surveys). Page1, Lines35-41
   - Diagnostic challenges. Page2, Lines2-5
   - Diagnosis (including other diagnoses considered). Page2, Lines2-5
   - Prognostic characteristics when applicable. Page2, Lines34-35
9. **Therapeutic Intervention**
   - Types of therapeutic intervention (pharmacologic, surgical, preventive). Page2, Lines7-8, Lines26-28
   - Administration of therapeutic intervention (dosage, strength, duration). Page2, Lines26-28
   - Changes in therapeutic interventions with explanations. Page2, Lines29-34
10. **Follow-up and Outcomes**
    - Clinician- and patient-assessed outcomes if available. Not available.
    - Important follow-up diagnostic and other test results. Page2, Lines29-34
    - Intervention adherence and tolerability. (How was this assessed?) Not available.
    - Adverse and unanticipated events. Page2, Lines34-35
11. **Discussion**
    - Strengths and limitations in your approach to this case. Page3, Lines7-9
    - Discussion of the relevant medical literature. Page3, Lines21-26
    - The rationale for your conclusions. Page2, Lines35-37; Page3, Lines28-30
    - The primary “take-away” lessons from this case report (without references) in a one paragraph conclusion. Page4, Lines17-24
12. **Patient Perspective** – The patient should share their perspective on the treatment(s) they received. Not available.
13. **Informed Consent** – The patient should give informed consent. (Provide if requested.) The patient had died, and the local ethics committee granted a waiver for informed consent. All personally identifiable patient information has been removed from this article.
